# Recent Advances in Photoelectrochemical Nitrate Reduction to Ammonia

**DOI:** 10.3390/ijms27010470

**Published:** 2026-01-01

**Authors:** Kaixin Zhu, Hefeng Zhang

**Affiliations:** Marine Engineering College, Dalian Maritime University, Linghai Road 1, Dalian 116026, China

**Keywords:** photoelectrochemical, nitrate reduction, ammonia, photocathode, solar energy

## Abstract

Ammonia, as an essential chemical, plays an indispensable role in both industry and agriculture. However, the traditional Haber–Bosch technique for ammonia synthesis suffers from high energy consumption and significant CO_2_ emissions. Therefore, developing an energy-efficient and eco-friendly method for ammonia production is imperative. Photoelectrochemical (PEC) nitrate reduction to ammonia has emerged as a promising green alternative, which utilizes renewable solar energy to convert nitrate into valuable ammonia, thereby contributing to nitrogen recycling and wastewater remediation. This review systematically summarizes recent advances in PEC nitrate reduction to ammonia, focusing on the rational design of efficient photocathodes with the development of semiconductor materials, cocatalysts, p–n junction and heterostructure strategies. Furthermore, the integration of photocathodes with photoanodes enables the assembly of bias-free PEC systems capable of simultaneously producing ammonia and value-added chemicals, demonstrating the potential for scalable solar-driven ammonia synthesis. The mechanistic studies and future research directions are also discussed. The review aims to offer valuable insights and promote the further development of PEC nitrate reduction to ammonia.

## 1. Introduction

Ammonia (NH_3_), the second most produced chemical worldwide, serves as a critical feedstock for pharmaceuticals, fertilizers, polymers, and other essential products [1,2,3]. As the primary downstream product of ammonia, fertilizers play an indispensable role in agricultural production [4,5]. The widespread application of fertilizers has significantly enhanced crop yields, thereby supporting rapid global population growth [6]. Furthermore, NH_3_ is regarded as a promising next-generation hydrogen carrier due to its ease of storage and transportation, high hydrogen content (17.6%), and carbon-free combustion characteristics [7,8,9]. Consequently, ammonia synthesis technologies have attracted extensive research interest. Currently, large-scale NH_3_ production predominantly relies on the conventional Haber–Bosch process, which converts N_2_ and H_2_ into NH_3_ under severe reaction conditions (400–500 °C and 20–30 MPa) [10,11,12,13]. However, such harsh operational requirements result in the ammonia synthesis process consuming approximately 1–2% of global energy supply and contributing about 1% of global CO_2_ emissions [14,15,16,17]. In the context of global efforts to reduce CO_2_ emission, the development of green ammonia synthesis technologies has become particularly important.

**Figure 1:**
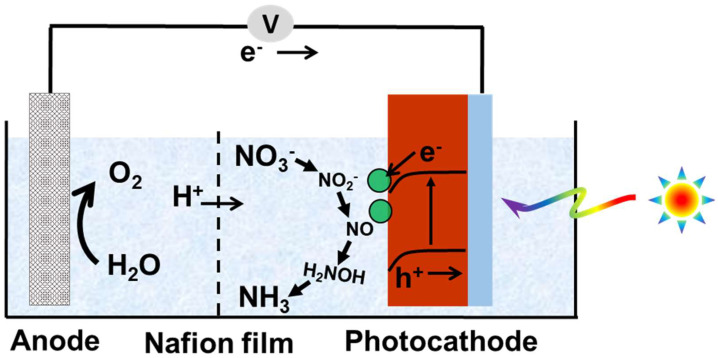
Schematic illustration of photoelectrochemical nitrate reduction to ammonia.

Nitrate (NO_3_^−^) is a widespread environmental pollutant, primarily originating from the discharge of nitrogen-containing wastewater and the excessive use of fertilizers [18,19,20]. The reduction of nitrate to ammonia not only mitigates the negative impacts of excess nitrate on ecosystems and the environment, but also enables the resource utilization of waste, thereby promoting the recycling of nitrogen element within the biosphere [21,22,23]. The photoelectrochemical (PEC) nitrate reduction to ammonia technology utilizes renewable solar energy to convert nitrate into valuable NH_3_, providing a feasible pathway for distributed green ammonia synthesis [24,25,26]. Similar to PEC water splitting for hydrogen production [27,28,29], the PEC nitrate reduction process involves three steps (Figure 1). Firstly, the semiconductor photocathode absorbs photons with energy greater than its bandgap, producing photogenerated electron-hole pairs. Subsequently, under an applied bias, the photogenerated electrons and holes are separated and directionally transported. The photogenerated electrons migrate to the surface of the photocathode, while the photogenerated holes are transferred to the anode through the external circuit. Finally, the electrons reaching the photocathode surface reduce nitrate to ammonia with the assistance of cocatalysts. The holes at the anode oxidize water to produce oxygen. Although the N=O bond in NO_3_^−^ has a low dissociation energy (204 kJ·mol^−1^) [30,31,32], the reduction of nitrate to ammonia is a complex multi-step reaction with eight electrons and nine protons (Table 1) [33,34,35]. This reaction proceeds through several intermediates (Figure 1), resulting in the formation of various by-products, such as NO_2_^−^, NO, and H_2_NOH, alongside the target product NH_3_ [36,37,38]. Therefore, research on PEC nitrate reduction to ammonia is still in its early stages. In recent years, growing interest in solar-driven ammonia synthesis via photoelectrochemical nitrate reduction has stimulated significant progress in this field, and several excellent reviews have been published [24,25,39]. However, a dedicated review that specifically and systematically summarizes photocathodes for PEC nitrate reduction to ammonia remains lacking.

In this review, we provide an overview of the recent advances in the development of photoelectrochemical nitrate reduction to ammonia. The review covers representative works on photocathodes with the employment of semiconductor materials, cocatalysts, p–n junctions and heterostructures. The possible mechanisms responsible for the improved performance of photocathodes are discussed. The bias-free systems for PEC nitrate reduction to NH_3_ are highlighted. We also provide perspectives on future research directions in this emerging field. Overall, this review aims to offer valuable insights for the development of solar-driven green ammonia synthesis technologies.

## 2. Photocathodes for PEC Nitrate Reduction to NH_3_

### 2.1. Antimony-Based Photocathodes

**Figure 2:**
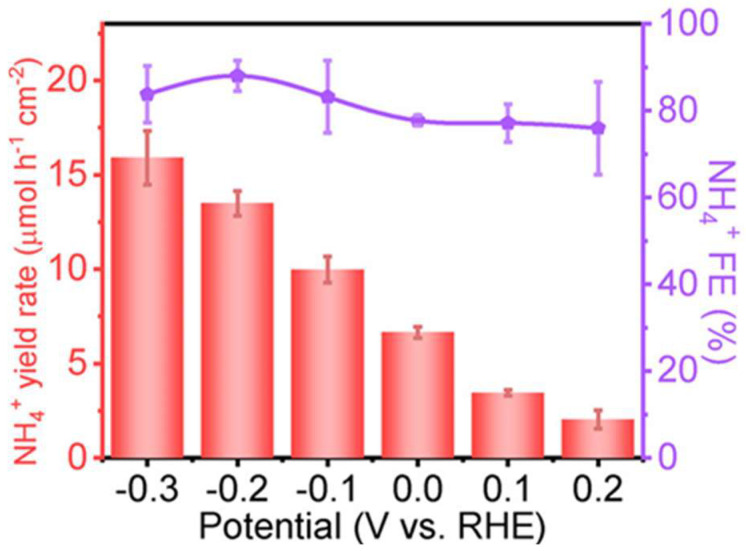
NH_4_^+^ Faradaic efficiencies and yield rates of CoCu/TiO_2_/Sb_2_Se_3_ in various applied potentials. The error bars denote the standard deviation of the Faradaic efficiencies and yield rates calculated from three independent samples. Reproduced with permission from [47]. Copyright WILEY-VCH Verlag GmbH & Co. KGaA, Weinheim, 2024.

Antimony selenide (Sb_2_Se_3_) is an important p-type semiconductor material owing to its narrow bandgap (~1.2 eV), low cost, large absorption coefficient, and high carrier mobility [40,41,42,43]. It can be used as a photocathode in PEC reactions such as PEC water splitting for hydrogen production [44,45,46]. Recently, Wang et al. reported an Sb_2_Se_3_-based photocathode for highly selective photoelectrochemical nitrate reduction to ammonia [47]. The Sb_2_Se_3_ nanorods were fabricated on fluorine-doped tin oxide (FTO) substrates via spin-coating and annealing, followed by the deposition of a TiO_2_ protective layer using atomic layer deposition. Finally, a CoCu co-catalyst was introduced on the surface via electrodeposition, forming a CoCu/TiO_2_/Sb_2_Se_3_ composite photocathode. The PEC nitrate reduction performance of the CoCu/TiO_2_/Sb_2_Se_3_ photocathode was evaluated in a 0.1 M KNO_3_ electrolyte containing 10 mM H_2_SO_4_ under AM 1.5 G (100 mW cm^−2^) illumination. The photocathode exhibited an onset potential of 0.43 V vs. reversible hydrogen electrode (RHE) and a photocurrent density of 4.75 mA·cm^−2^ at −0.3 V vs. RHE. As shown in Figure 2, within the potential range of −0.3 V to 0.2 V vs. RHE, the average Faradaic efficiency for NH_4_^+^ exceeded 75%. The highest NH_4_^+^ Faradaic efficiency of 88.01% was achieved at −0.2 V vs. RHE, with a corresponding NH_4_^+^ production rate of 13.49 μmol·h^−1^·cm^−2^. To further investigate the reaction mechanism, density functional theory (DFT) calculations were performed. The results indicated that Co plays a critical role in the CoCu co-catalyst. On the surface of CoCu/TiO_2_/Sb_2_Se_3_ photocathode, Cu sites serve as the main active centers for nitrate reduction to ammonia. The introduction of Co can improve the spin density of Cu and enhance the density of states near the Fermi level in the projected density of states (PDOS), thereby improving the PEC nitrate reduction activity of the CoCu/TiO_2_/Sb_2_Se_3_. In addition, the photocathode was tested in a simulated wastewater with low nitrate content to assess its performance under practical conditions. The simulated wastewater contained 3 mM NO_3_^−^, 0.07 M Cl^−^, and 0.05 M SO_4_^2−^. At −0.3 V vs. RHE, a Faradaic efficiency of 44% for NH_4_^+^ was still achieved, demonstrating the potential of the CoCu/TiO_2_/Sb_2_Se_3_ photocathode for solar-driven PEC nitrate reduction to ammonia in real wastewater environments.

Similar to Sb_2_Se_3_, Sb_2_S_3_ also exhibits a p-type property [48,49,50]. Sb_2_S_3_ is regarded as a promising photocathode for the efficient photoelectrochemical reduction of nitrate to ammonia due to a suitable bandgap of approximately 1.7 eV, a high light absorption coefficient and a long carrier diffusion length [51,52,53]. Wang et al. fabricated a CuSn/TiO_2_/Sb_2_S_3_ photocathode, which achieved a Faradaic efficiency for ammonia of 97.82% at 0.4 V vs. RHE, and an ammonia yield rate of 16.96 μmol·h^−1^·cm^−2^ at 0 V vs. RHE under AM 1.5 G (100 mW cm^−2^) illumination [54]. Moreover, the onset potential of the photocathode positively shifted to 0.62 V vs. RHE, and it demonstrated good stability over 4 h, with the ammonia yield rate increasing linearly. Structural characterizations revealed that the addition of Sn into the CuSn cocatalyst increases the lattice spacing of Cu and induces electron redistribution, with electron density transferring from Sn to Cu. This electron redistribution optimizes the adsorption of reactant NO_3_^−^ and enhances the hydrogenation of intermediates, thereby significantly accelerating the reaction kinetics of the PEC nitrate reduction reaction on the CuSn/TiO_2_/Sb_2_S_3_ photocathode and leading to high ammonia yield and Faradaic efficiency. Charge carrier kinetics studies further indicated that the CuSn/TiO_2_/Sb_2_S_3_ photocathode exhibits high charge separation efficiency and low carrier recombination rate, facilitating the rapid transfer of photogenerated electrons in the nitrate reduction reaction. DFT calculations demonstrated that the CuSn/TiO_2_/Sb_2_S_3_ surface exhibits a moderate adsorption energy for the intermediate, which ensures sufficient catalytic reaction time without hindering product desorption. This optimizes the reaction pathway and effectively suppresses competing side reactions such as the hydrogen evolution reaction (HER).

**Figure 3:**
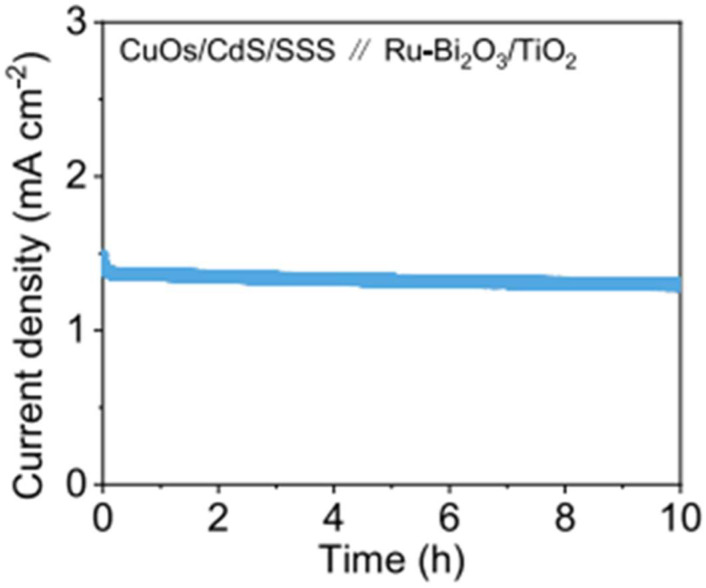
Unbiased stability for the CuOs/CdS/SSS||Ru-Bi_2_O_3_/TiO_2_ devices. Reproduced with permission from [60]. Copyright Springer Nature, 2025.

In photoelectrochemical nitrate reduction for ammonia production, the oxygen evolution reaction (OER) typically occurs at the anode. However, considering the high thermodynamic potential (1.23 V) of the water oxidation reaction and the limited economic value of oxygen [55,56], replacing OER with thermodynamically more favorable reactions that generate value-added products represents a promising alternative strategy [57,58,59]. Recently, Wang et al. developed a Sb_2_(S,Se)_3_-based photocathode for photoelectrochemical nitrate reduction to ammonia [60]. The photocathode featured a multilayer structure, with gradient selenium-doped Sb_2_(S,Se)_3_ as the light-absorbing layer, CdS as the electron transport layer, and TiO_2_ as the protective layer. A copper-osmium (CuOs) cocatalyst was further deposited onto the TiO_2_ layer to form the final CuOs/TiO_2_/CdS/Sb_2_(S,Se)_3_ (denoted as CuOs/CdS/SSS) photocathode. At 0 V vs. RHE, the CuOs/CdS/SSS photocathode achieves a photocurrent density of 5.6 mA·cm^−2^, with the onset potential shifting positively to 0.86 V vs. RHE. At 0.6 V vs. RHE, a Faradaic efficiency of 96.98% for ammonia is achieved. DFT calculations indicate that Os modification facilitates the provision of *H atoms, thereby enhancing the selectivity of ammonia. On the anode side, a Ru-Bi_2_O_3_/TiO_2_ photoanode was developed for the glycerol oxidation reaction. Under AM 1.5 G (100 mW·cm^−2^) illumination, the photoanode achieved a Faradaic efficiency of 42.9% and a selectivity of 59.2% for glycerol oxidation to dihydroxyacetone (DHA) at 0.6 V vs. RHE. For the three-carbon (C_3_) products (including glyceric acid, DHA, and glyceraldehyde), the total Faradaic efficiency reached 62.9% at 0.6 V vs. RHE. Fourier-transform infrared spectroscopy (FTIR) analysis revealed that Ru-Bi_2_O_3_/TiO_2_ favors the adsorption of intermediate hydroxyl groups, which promotes the formation of DHA. By coupling the CuOs/CdS/SSS photocathode with Ru-Bi_2_O_3_/TiO_2_ photoanode, a bias-free photoelectrochemical system was constructed. As shown in Figure 3, under AM 1.5 G (100 mW·cm^−2^) illumination, the system operates spontaneously, delivering a stable photocurrent density of approximately 1.4 mA·cm^−2^ without significant decay over 10 h of continuous reaction. This bias-free system simultaneously achieves efficient synthesis of ammonia and C_3_ products (such as DHA), with Faradaic efficiencies exceeding 90% and 77%, respectively.

**Figure 4:**
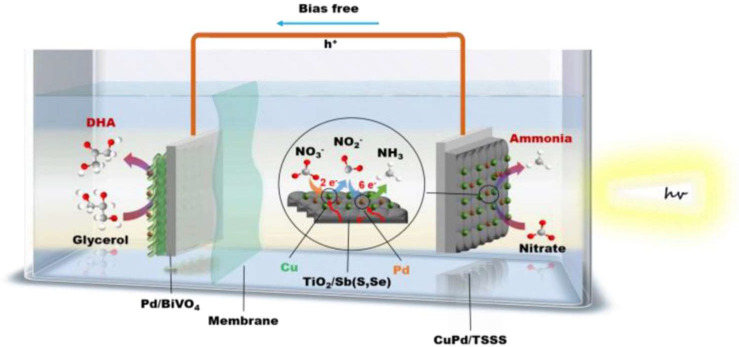
Schematic of a PEC cell fabricated by coupling the CuPd/TiO_2_/Sb_2_(S,Se)_3_ photocathode with the Pd/BiVO_4_ photoanode for unbiased production of NH_3_ and DHA. Reproduced with permission from [61]. Copyright WILEY-VCH Verlag GmbH & Co. KGaA, Weinheim, 2025.

Moreover, Wang et al. reported a bias-free system by coupling a CuPd/TiO_2_/Sb_2_(S,Se)_3_ photocathode with a Pd/BiVO_4_ photoanode to achieve nitrate reduction and glycerol oxidation (Figure 4) [61]. After 5 h of operation under AM 1.5 G (100 mW·cm^−2^) illumination, the accumulated yields of NH_3_ and DHA in this bias-free system reached 11.98 µmol·cm^−2^ and 20.19 µmol·cm^−2^, respectively. These studies demonstrate the considerable potential of photoelectrochemical systems to simultaneously produce ammonia and high-value chemicals. They provide a feasible avenue for green synthesis utilizing solar energy, nitrate-containing wastewater, and biomass byproducts, thereby contributing significantly to the advancement of sustainable energy and resource recycling.

### 2.2. Silicon

Silicon (Si) is extensively employed as a photocathode due to its excellent properties, such as narrow bandgap (1.12 eV), high earth abundance and suitable band alignment [62,63,64,65,66]. Gao et al. coated a p-type silicon nanowire array with an ultrathin TiO_2_ passivation layer by atomic layer deposition (ALD) method [67]. The oxygen defect in the TiO_2_ layer served as the adsorption site for nitrate and reduced the energy barriers of key steps in the nitrate reduction reaction. By varying the number of ALD cycles, the oxygen defect content was effectively modulated. The sample prepared with 50 cycles exhibited optimal performance for photoelectrochemical nitrate reduction to ammonia, achieving an NH_3_ production rate of 1074 μg h^−1^ cm^−2^ and a Faradaic efficiency of 94.3% for NO_3_^−^-to-NH_3_ conversion at −0.6 V vs. RHE.

**Figure 5:**
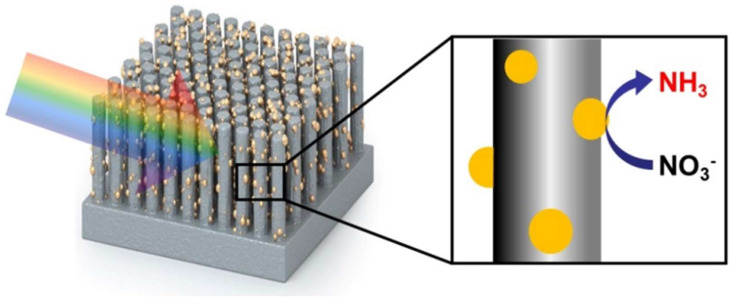
Schematic illustration of photoelectrochemical reduction of nitrate to ammonia using O_SiNW/Au. Reproduced with permission from [68]. Copyright WILEY-VCH Verlag GmbH & Co. KGaA, Weinheim, 2022.

Lee et al. reported an ordered silicon nanowire array photocathode decorated with Au nanoparticles (O_SiNW/Au) for the photoelectrochemical reduction of nitrate to ammonia (Figure 5) [68]. The O_SiNW/Au photocathode achieved a high Faradaic efficiency (FE) of 95.6% for NH_3_ at 0.2 V vs. RHE. It was demonstrated that the ordered silicon nanowire structure facilitated uniform distribution of Au nanoparticles cocatalyst and enhanced mass transport during the reaction. Furthermore, the inherent inactivity of both Si and Au surfaces toward the competing hydrogen evolution reaction contributed to the high Faradaic efficiency for ammonia generation.

Pan et al. fabricated a Co_0_._95_Ni_0_._05_-modified Si (Co_0_._95_Ni_0_._05_/Si) photocathode via photo-assisted electrodeposition for the photoelectrochemical nitrate reduction to ammonia [69]. The photocathode exhibited an onset potential of 0.42 V vs. RHE and achieved an ammonia yield rate of 2054 µg h^−1^ cm^−2^ with a Faradaic efficiency of 98.6% at −0.1 V vs. RHE. Furthermore, The Co_0_._95_Ni_0_._05_/Si photocathode demonstrated high durability during prolonged operation, maintaining the ammonia Faradaic efficiency above 90% and the stable average ammonia production rate. Structural characterization and theoretical calculations revealed that the Co_0_._95_Ni_0_._05_ cocatalyst was composed of a metallic Co core and a Ni-incorporated Co_3_O_4_ shell. The metallic Co core facilitates efficient electron transfer, yielding a high photovoltage, while the incorporation of Ni into Co_3_O4 enhances the reaction kinetics and ammonia selectivity.

Xiao et al. developed a copper nanoparticle-decorated silicon nanowire (Cu–Si NW) photocathode for the photoelectrochemical nitrate reduction to ammonia in acidic electrolyte [70]. The Si NW array was fabricated via metal-assisted chemical etching method. Cu nanoparticles were uniformly distributed on the silicon nanowires by a facile photo-deposition strategy. Under AM 1.5 G (100 mW cm^−2^) illumination, the Cu–Si NW photocathode exhibited an excellent performance for nitrate reduction to ammonia in 0.5 M H_2_SO4 electrolyte, achieving a positive onset potential of 0.3 V vs. RHE, a high photocurrent density of −34.29 mA cm^−2^, and a Faradaic efficiency of 97.03% for NH_4_^+^. Mechanistic studies revealed that the well-matched work functions and Fermi levels of Cu and silicon contributed to a favorable band alignment at the Cu–Si interface, thereby facilitating efficient charge transfer. In situ experiments and theoretical calculations further demonstrated that the Cu cocatalyst optimizes intermediate adsorption and promotes the protonation steps of NO_3_^−^. Moreover, the Cu–Si NW photocathode exhibited outstanding durability in simulated industrial wastewater treatment experiments, indicating its significant potential for practical applications.

Seo et al. constructed a crystalline silicon (c-Si) photocathode modified with Ni foil for the photoelectrochemical nitrate reduction reaction [71]. The Ni foil serves not only as an encapsulation layer to protect the c-Si from the electrolyte but also undergoes self-activation under alkaline conditions to form Ni(OH)_2_, which acts as an active catalyst for the nitrate reduction to ammonia. Under AM 1.5 G (100 mW cm^−2^) illumination, the photocathode exhibited an ammonia yield of 2468 μg cm^−2^ h^−1^ at –0.1 V vs. RHE, with a Faradaic efficiency of 85% for ammonia. Moreover, the photocathode demonstrated high durability, maintaining a stable photocurrent over multiple cycling tests. Experimental and theoretical studies revealed that the self-activated Ni(OH)_2_ effectively suppresses the competing hydrogen evolution reaction, thereby promoting the conversion of nitrate to ammonia. Furthermore, an all-back contact (ABC) c-Si photocathode was fabricated to achieve solar-driven ammonia synthesis under bias-free condition. The system remained stable over 51 h of continuous operation, delivering an ammonia production rate of 554 μg cm^−2^ h^−1^.

Benefiting from the well-established photovoltaic technology, commercial solar cells can serve as the light absorbers in photoelectrodes [72,73,74]. Amal et al. integrated a copper nano-structured top layer (Cu-NSTL) and Co(OH)_2_ nanosheets onto a commercial Si solar cell to construct a Si/Cu-NSTL/Co(OH)_2_ ternary photocathode [75]. The photocathode exhibited high performance in photoelectrochemical nitrate reduction to ammonia, demonstrating an onset potential of 1.0 V vs. RHE. Across the applied potential range from 0.6 to 0 V vs. RHE, the ammonia production rate increased from 22.3 to 106.6 μmol·h^−1^·cm^−2^, while the Faradaic efficiency consistently remained above 90%. Moreover, the photocathode demonstrated good structural integrity and catalytic stability. Mechanistic studies revealed a synergistic effect between Cu-NSTL and Co(OH)_2_, which enhanced the transport of photogenerated electrons, promoted water dissociation, and facilitated the deoxygenation and hydrogenation of reactants/intermediates, thereby enabling highly efficient and selective ammonia production. Furthermore, a large-scale “artificial leaf” photoelectrochemical device was developed to simultaneously achieve nitrate reduction to ammonia and oxidation of biomass-derived glycerol to formate under bias-free conditions. Outdoor testing confirmed the promising practicality of this system.

### 2.3. Gallium Nitride

**Figure 6:**
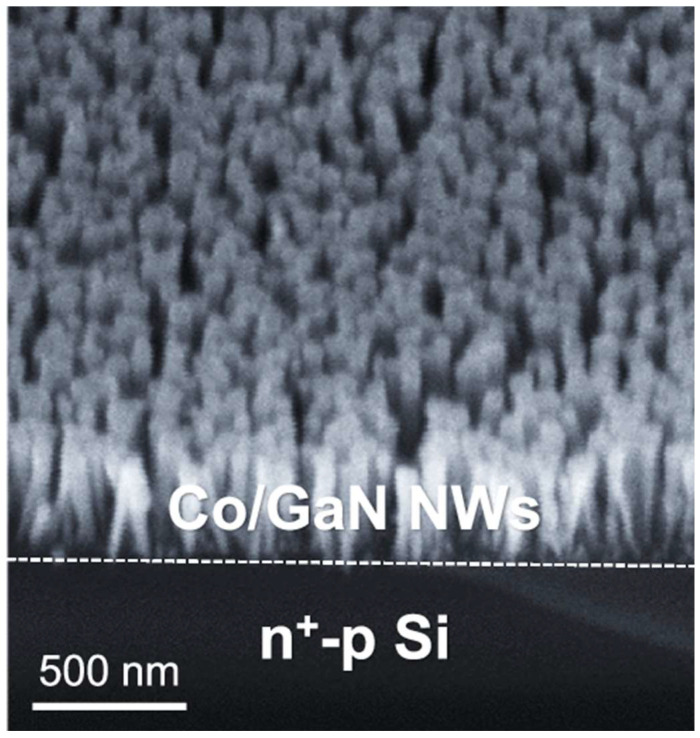
45 degree-tilt-view SEM image of Co/GaN/Si. Reproduced with permission from [80]. Copyright Springer Nature, 2025.

Gallium nitride (GaN) is a chemically stable semiconductor widely used in optoelectronic devices [76,77,78]. Mi et al. constructed an Au/GaN/Si photocathode by vertically growing GaN nanowires on an n+-p Si substrate via plasma-assisted molecular beam epitaxy, followed by photodeposition of Au nanoclusters on the surface [79]. Experimental data and theoretical calculations revealed that NO_3_^−^ is first adsorbed and reduced to NO_2_^−^ on the GaN surface, after which NO_2_^−^ migrates to adjacent Au nanoclusters for further hydrogenation to NH_3_. By optimizing the size and surface coverage of the Au nanoclusters, the performance for photoelectrochemical conversion of nitrate to ammonia was improved, delivering a Faradaic efficiency for ammonia of 91.8% at −0.4 V vs. RHE and an ammonia yield rate of 131.1 μmol·cm^−2^·h^−1^ at −0.8 V vs. RHE, with no apparent degradation over 8 h. Furthermore, Mi et al. fabricated Co/GaN/Si and Ni/GaN/Si photocathodes by employing transition metals Co and Ni as cocatalysts, respectively [80]. The Scanning electron microscopy (SEM) image showed GaN NWs with lengths of about 400 nm (Figure 6). The Co/GaN/Si and Ni/GaN/Si photocathodes exhibited the Faradaic efficiency for ammonia production close to 100% at potentials more positive than 0 V vs. RHE. In terms of ammonia yield rate, the Co/GaN/Si photocathode achieved 166.7 µmol h^−1^ cm^−2^ at −0.7 V vs. RHE, while the Ni/GaN/Si photocathode reached 201.6 µmol h^−1^ cm^−2^ at −0.4 V vs. RHE.

### 2.4. Oxides

P-type BiVO_4_ is a promising photocathode material [81,82,83]. Fan et al. prepared a p-BiVO_4_ photocathode modified with amorphous metal oxide CoFeMnO (denoted as CoFeMnO/BiVO_4_) [84]. The CoFeMnO cocatalyst promoted the carrier density, NO_3_^−^ adsorption, and electron transfer kinetics, thereby boosting the photoelectrochemical nitrate reduction to ammonia. At −0.1 V vs. RHE, the CoFeMnO/BiVO_4_ photocathode achieved a current density of −0.36 mA cm^−2^, an NH_3_ production rate of 17.82 µg h^−1^ cm^−2^, and a Faradaic efficiency of 32.8%. Moreover, the photocathode exhibited high stability, maintaining consistent current without obvious degradation over 12 h. In addition, Fan et al. reported a ZnIn_2_S_4_/BiVO_4_ heterostructure with the abundant zinc vacancies (V_Zn_) [85]. The V_Zn_ induces the formation of “frustrated Lewis pairs (FLPs)”. The FLPs promote the adsorption and activation of NO_3_^−^ ions, while the V_Zn_ sites suppress charge carrier recombination. The ZnIn_2_S_4_/BiVO_4_ photocathode achieved an ammonia production rate of 29.95 μg h^−1^ cm^−2^ and a Faradaic efficiency of 37.2% at −0.1 V vs. RHE.

Cu_2_O is a typical p-type semiconductor with a bandgap of 2.0 eV, which has been widely used in solar energy conversion [86,87,88]. However, the application of Cu_2_O in photoelectrochemical reactions has been limited by its photocorrosion, often necessitating protection layers to enhance its stability [89,90,91]. Notably, Choi et al. reported that the surface of pristine Cu_2_O exhibits catalytic activity toward the photoelectrochemical nitrate reduction, which kinetically suppresses the photocorrosion of Cu_2_O without requiring additional cocatalysts or protection layers [92]. The Cu_2_O photocathode selectively reduces nitrate to nitrite with a Faradaic efficiency exceeding 85%. In contrast to nitrate reduction, the photoelectrochemical reduction of nitrite on the Cu_2_O photocathode is slower and fails to suppress photocorrosion effectively. The reaction primarily yields ammonia with a Faradaic efficiency of about 50%. Furthermore, Hou et al. employed TiO_2_ and Al-doped ZnO (AZO) as protective layers to fabricate a TiO_2_/AZO/Cu_2_O/Au photocathode [93]. By coupling with a CdS/CdIn_2_S_4_ photoanode, the integrated system simultaneously achieved photoelectrochemical benzyl alcohol oxidation and nitrite reduction to ammonia, with a maximum Faradaic efficiency exceeding 98%.

Titanium dioxide (TiO_2_) is the most commonly used semiconductor in photoelectrochemistry [94,95]. Silveira et al. reported a NiO/Au plasmon/TiO_2_ composite for photo-assisted electrocatalytic nitrate reduction to ammonia [96]. Their study revealed that amorphous TiO_2_ markedly promotes the conversion of nitrate to nitrite, elevating the nitrite concentration in solution by nearly 50% and improving the corresponding Faradaic efficiency by 10%. In contrast, the rutile phase TiO_2_ primarily facilitates the subsequent reduction of nitrite to ammonia, increasing the Faradaic efficiency by 30%.

### 2.5. Cu_2_ZnSnS_4_

**Figure 7:**
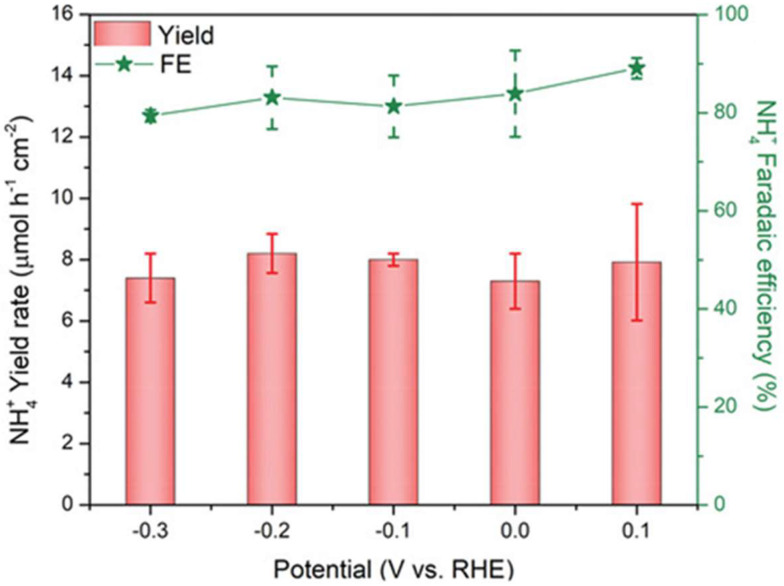
NH_4_^+^ Faradaic efficiency and yield rate of TiOₓ-250/CdS/CZTS in different applied potentials; the error bars denote the standard deviation (SD) of the Faradaic efficiencies and yield rates calculated from three independent samples. Reproduced with permission from [100]. Copyright WILEY-VCH Verlag GmbH & Co. KGaA, Weinheim, 2022.

Cu_2_ZnSnS_4_ (CZTS) semiconductor has emerged as a promising photocathode material due to its excellent light absorption, environmental compatibility, and earth-abundant availability [97,98,99]. Amal et al. developed a CZTS-based photocathode, in which CZTS serves as the light-absorbing layer, CdS buffer layer forms a p–n junction to facilitate charge separation, and TiOₓ layer is modified on the surface as a cocatalyst [100]. By optimizing the preparation temperature of the TiOₓ layer, the optimal photocathode (TiOₓ-250/CdS/CZTS) was obtained. The photocathode exhibits an onset potential of 0.38 V vs. RHE, a Faradaic efficiency for ammonia of 89.1% at 0.1 V vs. RHE, and a maximum production rate of 8.21 µmol·h^−1^·cm^−2^ at −0.2 V vs. RHE (Figure 7). It also demonstrates good stability, retaining over 80% of initial photocurrent density after 5 h reaction. Experimental and theoretical analyses reveal that the Ti^3+^ species in the TiOₓ layer enhance the adsorption of NO_3_^−^, thereby suppressing the formation of the by-product NO_2_^−^ and improving the selectivity toward ammonia. Furthermore, the TiOₓ-250/CdS/CZTS photocathode sustains ammonia production even in simulated wastewater, achieving a Faradaic efficiency of 64.9% at −0.38 V vs. RHE and a production rate of 6.54 µmol·h^−1^·cm^−2^.

### 2.6. Organic Semiconductor Photocathodes

**Figure 8:**
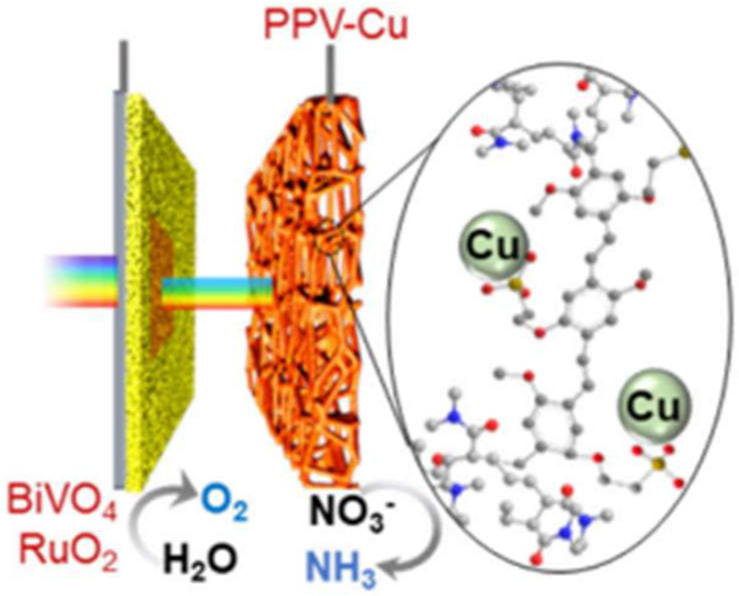
Schematic illustration of PPV-Cu and BiVO_4_ tandem system. Reproduced with permission from [104]. Copyright American Chemical Society, 2024.

Compared with inorganic materials, organic polymer semiconductors exhibit highly tunable optoelectronic properties, enabling the construction of diverse photoelectrodes for the targeted reactions [101,102,103]. Recently, Shan et al. designed a photoconductive supramolecular network based on poly(2-methoxy-5-propyloxysulfonate phenylene vinylene) (PPV) for photoelectrochemical reduction of nitrate to ammonia [104]. The PPV network with an ordered porous structure was decorated by a Cu cocatalyst via site-selective deposition to form a PPV-Cu photocathode. Under AM 1.5 G illumination (100 mW cm^−2^), the photocathode delivered an average photocurrent density of 8.5 mA cm^−2^, with a Faradaic efficiency of 95% for nitrate reduction to ammonia. Furthermore, as shown in Figure 8, a tandem photoelectrochemical system was fabricated by integrating PPV–Cu photocathode with a BiVO_4_–RuO_2_ photoanode, which can simultaneously realize nitrate reduction to ammonia and water oxidation to oxygen. The tandem system achieved high Faradaic efficiencies of 95–98% for both NH_3_ and O_2_, with photocurrent and product yields 10 times higher than the single-junction BiVO_4_–RuO_2_.

**Figure 9:**
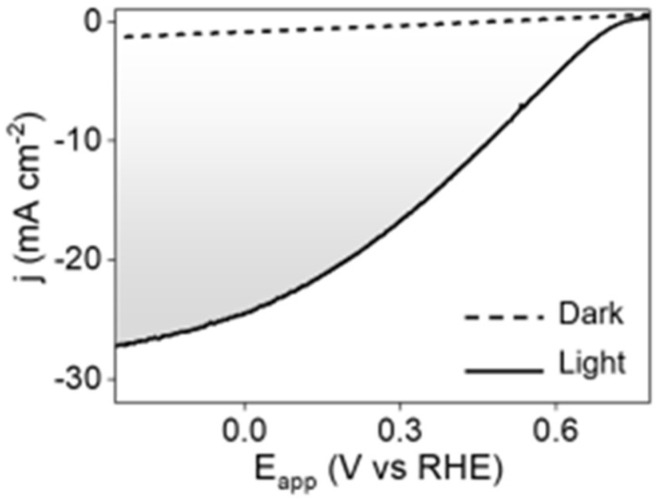
Current density (j) of OPN-CuCo under AM 1.5 G (100 mW cm^−2^) irradiation (solid line) and under dark (dashed line) in aqueous KNO_3_ (1.0 M) at pH 4.5. Scan rate: 50 mVs^−1^. E_app_: applied bias. Reproduced with permission from [105]. Copyright WILEY-VCH Verlag GmbH & Co. KGaA, Weinheim, 2025.

In order to improve the performance of organic photoelectrodes, Shan et al. proposed an organic p–n junction (OPN) strategy to prepare polymer-based photocathode for solar-driven ammonia production [105]. The p-type poly(3,4-ethylenedioxythiophene) (PEDOT) and n-type perylene diimide (PDI) were assembled in a covalent framework to fabricate the OPN photocathode. Surface photovoltage mapping and atomic force microscopy confirmed that photogenerated electrons and holes in the OPN photocathode were spatially separated in the PDI and PEDOT regions, respectively, with a separation distance of approximately 36 nm. The CuCo cocatalyst was subsequently incorporated into the OPN matrix via in situ electrochemical deposition, yielding the OPN–CuCo photocathode. As shown in Figure 9, under AM 1.5 G (100 mW cm^−2^) irradiation, the OPN-CuCo photocathode achieved a photocurrent density of 27 mA cm^−2^ at 0.10 V vs. RHE for photochemical nitrate reduction to NH_3_. It exhibited a Faradaic efficiency of 96% for NH_3_ production. In situ spectroscopy and scanning electrochemical microscopy revealed that the Co component in the CuCo catalyst facilitates the protonation of the intermediate. Density functional theory calculations further indicated that the introduction of Co lowers the energy barrier, thereby enhancing the selectivity toward ammonia. To construct a bias-free photoelectrochemical system, the OPN–CuCo photocathode was coupled with a silicon solar cell in a flow-cell system. The bias-free system delivered a photocurrent density of 57 mA cm^−2^. Moreover, the generated ammonia was collected in the form of NH_4_Cl crystals via an air-stripping technique, achieving an overall collection efficiency of 87%.

Organic molecules, such as copper phthalocyanine (CuPc), show a wide potential for solar energy conversion [106,107,108]. Shi et al. reported a CuPc/CeO_2_ heterostructure photocathode for photoelectrochemical nitrate reduction to ammonia [109]. Under light irradiation, photogenerated electrons in the lowest unoccupied molecular orbital (LUMO) of CuPc are transferred to the conduction band of CeO_2_, quickly captured by the abundant oxygen vacancies on the CeO_2_ surface. These trapped electrons subsequently react with the adsorbed NO_3_^−^ to produce NH_3_. Photoelectrochemical tests demonstrated that the CuPc/CeO_2_ photocathode achieves an ammonia production rate of 1.16 µmol·h^−1^·cm^−2^ with a Faradaic efficiency of 33% at −0.6 V vs. RHE. Moreover, the photocathode exhibits acceptable stability, maintaining its NH_3_ yield rate without significant degradation over 5 cycling tests.

### 2.7. Organic–Inorganic Hybrid Perovskites

Organic-inorganic hybrid perovskites have shown great promise for highly efficient solar energy conversion due to their excellent properties, such as high extinction coefficient, tunable bandgap, and high charge carrier mobility [110,111,112]. Recently, Jang et al. fabricated a Cs_0_._05_(FA_0_._83_MA_0_._17_)_0_._95_Pb(Br_0_._17_I_0_._83_)_3_ perovskite-based photocathode with a Ru-loaded titanate nanosheet (TiNS) cocatalyst (Ru@TiNS) for highly efficient and selective nitrate reduction to NH_3_ [113]. To address the instability of perovskite materials in aqueous environments, a protective layer composed of Ni foil and Field’s metal was introduced, which effectively prevented electrolyte penetration and facilitated charge transport. Under AM 1.5 G (100 mW cm^−2^) illumination, the Ru@TiNS/Ni/perovskite photocathode exhibited an onset potential of 1.5 V vs. RHE and a Faradaic efficiency of 93.7% for NH_3_ production at 0.62 V vs. RHE. The photocurrent density remained stable without significant degradation over 24 h of continuous reaction. Furthermore, glycerol oxidation was carried out on a Pt-loaded TiNS electrocatalyst (Pt@TiNS). By coupling the perovskite-based photocathode with the glycerol oxidation anode, a bias-free photoelectrochemical system was constructed. Under AM 1.5 G (100 mW cm^−2^) illumination, the bias-free system delivered a photocurrent density of 21.2 mA cm^−2^ and an ammonia production rate of 1744.9 µg cm^−2^ h^−1^ with an NH_3_ Faradaic efficiency of 99.5%. Simultaneously, glycerol was oxidized to glyceric acid and lactic acid, with a total Faradaic efficiency of 98.1%. The system maintained stable performance over 24 h of continuous operation, demonstrating great potential for sustainable ammonia synthesis and valorization of biomass-derived compounds.

The performance of representative photocathodes for photoelectrochemical nitrate reduction to ammonia is exhibited in Table 2.

## 3. Conclusions and Perspectives

Photoelectrochemical nitrate reduction to ammonia provides an ideal method for green ammonia synthesis. The development of efficient photocathode materials is essential for achieving solar-driven green ammonia production. Considerable efforts have been devoted to investigating various semiconductor materials, and significant progress has been achieved. For instance, several p-type semiconductors have been employed to fabricate photocathodes for nitrate reduction to ammonia, and their performance has been enhanced through strategies such as cocatalyst modification, construction of p–n junctions, or heterostructures. The integration of photocathodes with photoanodes enables the fabrication of bias-free photoelectrochemical systems for nitrate reduction to ammonia. Although these preliminary results are encouraging, the current efficiency of ammonia synthesis is still low. This can be attributed to the multi-electron and multi-proton transfer processes involved in nitrate reduction, which lead to complex reaction pathways and a tendency for by-product formation. In addition, the practical application of PEC nitrate reduction to ammonia is primarily constrained by several intertwined bottlenecks. Intrinsic material limitations, including inefficient charge separation, limited broad-spectrum light absorption, and insufficient long-term stability of semiconductor photocathodes, remain central challenges. Concurrently, system-level design hurdles, such as achieving high energy efficiency and stability under unbiased operation, scaling up device architectures, and efficiently integrating photocathodes with selective photoanodes for value-added oxidation reactions, are equally critical.

To improve the ammonia yield and facilitate practical application, future research could focus on the following aspects: (i) developing novel photocathode materials with suitable band gaps for broad-spectrum absorption, high charge carrier mobility, and appropriate band energy levels; (ii) enhancing ammonia production rate by suppressing photogenerated charge carrier recombination through cocatalyst modification and interface engineering; (iii) gaining in-depth insight into the reaction mechanism of photoelectrochemical nitrate reduction to ammonia via in situ characterization techniques, thereby guiding the rational design and performance optimization of photocathodes; (iv) employing artificial intelligence (e.g., machine learning and high-throughput screening) integrated with first-principles calculations to explore complex reaction networks and discover high-performance semiconductor materials with excellent properties from vast candidate material libraries, thus reducing reliance on trial-and-error experimentation and enhancing efficiency in material research and development; and (v) Coupling new materials with scalable reactor architectures to realize practical implementation of PEC nitrate reduction to ammonia.

## Figures and Tables

**Table 1:** Reaction pathway for nitrate reduction to ammonia mediated by electron transfer.

Reaction Steps	E^Ɵ^ vs. Standard Hydrogen Electrode (SHE)
${NO}_{3(ad)}^{-}$ $+e^{-}\to {NO}_{3(ad)}^{2-}$	−0.89 V
${NO}_{3(ad)}^{2-}$ $+2H^{+}\to {NO}_{2\left(ad\right)}+H_{2}O$	—
${NO}_{2(ad)}+e^{-}\to {NO}_{2(ad)}^{-}$	1.04 V
${NO}_{2(ad)}^{-}+e^{-}\to {NO}_{2(ad)}^{2-}$	−0.47 V
${NO}_{2(ad)}^{2-}+H_{2}O\to {NO}_{\left(ad\right)}+2{OH}^{-}$	—
${NO}_{\left(ad\right)}+H^{+}+e^{-}\to {HNO}_{\left(ad\right)}$	−0.78 V
${HNO}_{\left(ad\right)}+H^{+}+e^{-}\to H_{2}{NO}_{\left(ad\right)}$	0.52 V
$H_{2}{NO}_{\left(ad\right)}+H^{+}+e^{-}\to H_{2}{NOH}_{\left(ad\right)}$	0.90 V
$H_{2}NOH+2H^{+}+2e^{-}\to {NH}_{3}+H_{2}O$	0.42 V

**Table 2:** Summary of recently representative photocathodes used in the photoelectrochemical nitrate reduction to ammonia.

Photocathode	Onset Potential (vs. RHE)	Yield Rate of NH_3_	Faradaic Efficiency of NH_3_	Stability	References
CoCu/TiO_2_/Sb_2_Se_3_	0.43 V	15.91 μmol h^−1^ cm^−2^ at −0.3 V vs. RHE	88.01% at −0.2 V vs. RHE	4 h	[47]
CuSn/TiO_2_/Sb_2_S_3_	0.62 V	16.96 μmol h^−1^ cm^−2^ at 0 V vs. RHE	97.82% at 0.4 V vs. RHE	4 h	[54]
CuOs/TiO_2_/CdS/Sb_2_(S,Se)_3_	0.86 V	19.87 μmol h^−1^ cm^−2^ at 0.1 V vs. RHE	96.98% at 0.6 V vs. RHE	12 h	[60]
CuPd/TiO_2_/Sb_2_(S,Se)_3_	0.83 V	14.5 μmol h^−1^ cm^−2^ at 0 V vs. RHE	94.6% at 0.8 V vs. RHE	6 h	[61]
Si@TiO_2_	—	63.17 umol h^−1^ cm^−2^ at −0.6 V vs. RHE	94.3% at −0.6 V vs. RHE	10 h	[67]
O_SiNW/Au	0.3 V	0.41 umol h^−1^ cm^−2^ at 0.1 V vs. RHE	95.6% at 0.2 V vs. RHE	8 h	[68]
Co_0_._95_Ni_0_._05_/Si	0.42 V	120.82 umol h^−1^ cm^−2^ at −0.1 V vs. RHE	98.6% at −0.1 V vs. RHE	6 h	[69]
Cu–Si NW	0.3 V	65.91 μmol h^−1^ cm^−2^ at −0.6 V vs. RHE	97.03% at −0.4 V vs. RHE	36 h	[70]
Ni(OH)_2_@Ni foil/c-Si	0.69 V	145.1 umol h^−1^ cm^−2^ at −0.1 V vs. RHE	85% at −0.1 V vs. RHE	5 h	[71]
Si/Cu-NSTL/Co(OH)_2_	1.0 V	106.6 μmol h^−1^ cm^−2^ at 0 V vs. RHE	nearly 100% at 0 V vs. RHE	10 h	[75]
Au/GaN/Si	−0.2 V	131.1 μmol h^−1^ cm^−2^ at −0.8 V vs. RHE	91.8% at −0.4 V vs. RHE	8 h	[79]
Co/GaN/Si or Ni/GaN/Si	0.3 V	201.6 μmol h^−1^ cm^−2^ at −0.4 V vs. RHE	99% at 0.2 V vs. RHE	10 h	[80]
CoFeMnO/BiVO_4_	—	1.04 umol h^−1^ cm^−2^ at −0.1 V vs. RHE	32.8% at −0.1 V vs. RHE	12 h	[84]
ZnIn_2_S_4_/BiVO_4_	—	1.76 μmo h^−1^ cm^−2^ at −0.1 V vs. RHE	37.2% at −0.1 V vs. RHE	13 h	[85]
Cu_2_O	0.7 V	—	15%	5 h	[92]
TiO_2_/AZO/Cu_2_O/Au	—	—	98.1%	1 h	[93]
TiOₓ/CdS/CZTS	0.38 V	8.21 μmol h^−1^ cm^−2^ at −0.2 V vs. RHE	89.1% at 0.1 V vs. RHE	5 h	[100]
PPV-Cu	—	—	95%	—	[104]
OPN–CuCo	—	—	96%	—	[105]
CuPc/CeO_2_	—	1.16 umol h^−1^ cm^−2^ at −0.6 V vs. RHE	33% at −0.6 V vs. RHE	2 h	[109]
Ru@TiNS/Ni/ Cs_0_._05_(FA_0_._83_MA_0_._17_)_0_._95_Pb(Br_0_._17_I_0_._83_)_3_	1.5 V	—	93.7% at 0.62 V vs. RHE	24 h	[113]

## Data Availability

No new data were created or analyzed in this study. Data sharing is not applicable.
